# The Role of Sex Hormone Receptors in the Squamous Cell Carcinoma of the Uterine Ectocervix: A Review and Future Directions

**DOI:** 10.3390/diagnostics16152424

**Published:** 2026-07-31

**Authors:** Mun-Kun Hong, Dah-Ching Ding

**Affiliations:** 1 Department of Obstetrics and Gynecology, Hualien Tzu Chi Hospital, Buddhist Tzu Chi Medical Foundation, Tzu Chi University, Hualien 970, Taiwan; jeff06038@gmail.com; 2 Institute of Medical Sciences, Tzu Chi University, Hualien 970, Taiwan

**Keywords:** cervical cancer, estrogen receptor α, progesterone receptor, stromal, HPV, sex hormone, tumor microenvironment, carcinogenesis, diagnostics

## Abstract

Cervical cancer (CxCa) remains a major global gynecological malignancy causally linked to high-risk human papillomavirus (HPV) infection. However, HPV alone is insufficient for carcinogenesis; sex hormones and their receptors serve as essential cofactors. This review aims to synthesize current knowledge on the role of cervical histoarchitecture and hormonal microenvironments in ectocervical squamous cell carcinoma (SCC), and to explore how receptor-mediated signals contribute to malignant transformation and metastatic spread. The review examines the expression patterns of estrogen receptor α (ERα) and progesterone receptor (PR) in the uterine cervix, which is a hormone-responsive tissue. It discusses how these receptors fluctuate throughout the menstrual cycle and evaluates evidence from HPV-transgenic mouse models and human tissue analyses. The findings converge on a compelling model: stromal ERα drives pro-carcinogenic paracrine signaling, epithelial PR suppresses neoplastic transformation, and stromal PRB confers antimetastatic protection. These insights highlight the complex interplay between hormonal signals and SCC development in the ectocervix. Future therapeutic exploitation of these mechanistic insights—including selective estrogen receptor modulators, selective progesterone receptor modulators, and PR status-guided chemoprevention—holds considerable promise. This review provides a coherent framework for translating laboratory findings into clinical diagnostics and targeted interventions.

## 1. Introduction

Cervical cancer (CxCa) is the fourth most common cancer among women worldwide, with an estimated 660,000 new cases and 350,000 deaths reported annually [1]. Despite the availability of prophylactic human papillomavirus (HPV) vaccines and widespread cytologic screening programs, cervical cancer remains a leading cause of cancer-related mortality in low- and middle-income countries [2]. Persistent infection with high-risk HPV genotypes, particularly HPV16 and HPV18, is the primary etiological driver [3]. However, most HPV-infected women never develop invasive disease, confirming that viral oncogenes alone are insufficient for malignant transformation [4].

Epidemiological evidence has long implicated sex hormones as key cofactors in cervical carcinogenesis. Age-specific incidence curves demonstrate that ectocervical squamous cell carcinoma (SCC) risk rises after menarche, peaks around menopause, and then declines—a pattern that parallels cumulative estrogen exposure [5]. Moreover, prolonged oral contraceptive use (≥5 years) elevates cervical cancer risk fourfold in HPV-positive women, and high parity is an independent risk factor, both associated with sustained elevations of estrogen and progesterone [6]. Postmenopausal women rarely develop new-onset cervical cancer, underscoring the hormonal dependence of cervical carcinogenesis [7].

The stroma of the uterine cervix is covered by squamous epithelium in the vagina (ectocervix) and by columnar epithelium in the cervical canal (endocervix), the two types of epithelium meet at the squamocolumnar junction, and the position of the squamocolumnar junction varies according to age, from childhood to puberty and adulthood [8]. The majority of cervical cancers are SCC and that adenocarcinomas make up most of the remainder [9].

The cervix is a hormonally responsive tissue derived from the Müllerian duct, expressing both estrogen receptor alpha (ERα) and PR in a compartment-specific and cycle-dependent manner [10]. Early studies measuring these receptors in cervical tissues were limited by small sample sizes and antibodies that could not discriminate between receptor isoforms [11]. Advances in HPV transgenic mouse models and high-resolution immunohistochemistry (IHC) have elucidated that the critical determinants of hormonal influence on cervical carcinogenesis reside not in the neoplastic epithelium—which progressively loses receptor expression—but in the surrounding stromal compartment [12,13].

Building on our previously reported cohort data establishing the prognostic significance of stromal ERα and PRB expression [7], the present review extends these findings by situating them within their developmental origin, physiological regulation across the menstrual cycle, and broader translational context, thereby providing a mechanistic framework that was outside the scope of the original cohort analysis.

This review focuses specifically on SCC arising from the ectocervix, the predominant histological subtype of cervical cancer; discussion of adenocarcinoma and of endocervical pathology, which involve distinct cellular origins and carcinogenic pathways, is beyond the scope of this review.

This review comprehensively reviews/synthesizes: (1) the embryological origin and histological organization of the cervix from the Müllerian duct; (2) physiological fluctuations in ERα and PR expression across the menstrual cycle; (3) epidemiological associations between sex hormones and cervical cancer; (4) the carcinogenic role of stromal ERα; (5) the anti-transformation role of epithelial PR; and (6) the antimetastatic role of stromal PRB. Finally, we discuss future research directions and the translational potential of targeting sex hormone receptors as a novel diagnostic and therapeutic strategy in CxCa.

### Search Strategy

Relevant literature was identified through searches of PubMed/MEDLINE and Google Scholar for articles published between inception and April 2026, encompassing both foundational mechanistic studies and recent literature. Search terms were combined using Boolean operators and included: (“cervical cancer” OR “cervical neoplasia” OR “cervical carcinogenesis”) AND (“estrogen receptor” OR “ERα” OR “progesterone receptor” OR “PR” OR “PRA” OR “PRB”) AND (“stroma” OR “stromal” OR “epithelium” OR “epithelial” OR “tumor microenvironment”), supplemented by targeted searches combining these terms with “HPV,” “Müllerian duct,” “menstrual cycle,” “SERM,” “medroxyprogesterone,” and “menopause.” Reference lists of retrieved articles, including key mouse-model studies from the Lambert/Chung/Korach research groups, were hand-searched to identify additional relevant publications. Articles were included if they reported original data or synthesized evidence on sex hormone receptor expression, regulation, or function in cervical tissue or HPV-associated cervical neoplasia, in either human specimens or validated animal models. Non-English-language articles, conference abstracts without full-text availability, and studies not addressing ERα/PR biology in the cervix were excluded. Given the narrative and mechanistic-synthesis nature of this review, no formal PRISMA-based systematic screening or quality-scoring protocol was applied; the aim was comprehensive coverage of the mechanistic and clinical literature relevant to the stromal–epithelial hormone receptor model, rather than systematic effect-size pooling.

## 2. The Development of the Müllerian Duct and Cervix

The uterine cervix arises from the Müllerian (paramesonephric) ducts, paired embryonic structures that form alongside the mesonephric (Wolffian) ducts in both sexes [14]. In the absence of anti-Müllerian hormone, which is secreted by fetal Sertoli cells to induce duct regression in males, Müllerian ducts persist and differentiate in females into the oviducts, uterus, cervix, and upper vagina [12]. Müllerian duct development proceeds through three sequential phases—initiation, invagination, and elongation—each governed by distinct molecular signals [14].

The initiation phase involves the specification of coelomic epithelial cells adjacent to the mesonephros. Bone morphogenetic protein (BMP) signaling activates the transcription factor PAX2, which subsequently engages the FGF/RAS/ERK pathway to upregulate LIM1, facilitating apical constriction and tubulogenesis [15]. WNT4 and WNT9b are critical for the caudal elongation of the Müllerian duct rudiment, with loss-of-function mutations in these genes causing reproductive tract aplasia [16]. Additional transcription factors, including EMX2, HOXA13, and FOXA2, govern regional identity along the craniocaudal axis, ensuring appropriate differentiation of each Müllerian segment [14].

The cervix specifically derives from the caudal segment of the Müllerian duct, where it fuses with the urogenital sinus to form the cervicovaginal junction [17]. This junction marks the transformation zone, a metaplastic transition zone between the endocervical columnar epithelium and the ectocervical stratified squamous epithelium. The transformation zone is enriched with reserve cells that are particularly susceptible to HPV infection and oncogenic transformation [18]. The mesenchyme surrounding the Müllerian epithelium plays a critical inductive role during organogenesis: stromal-epithelial cross-talk, mediated in part by estrogen signaling through stromal ERα, orchestrates region-specific epithelial differentiation [19]. This developmental blueprint—where stromal paracrine signals govern epithelial behavior—persists in adult cervical physiology and is reactivated during carcinogenesis [20].

Understanding this developmental anatomy is crucial because it establishes the histological and molecular foundation upon which sex hormone receptor biology operates. The distinct expression of ERα and PR in the stromal versus epithelial compartments of the adult cervix reflects the embryonic pattern of hormonally responsive mesenchyme directing the overlying epithelium [7]. Disruptions in Müllerian duct development, including congenital uterine anomalies, have also been associated with altered hormonal receptor expression and elevated gynecologic cancer risk, further linking embryological programming to adult oncogenic susceptibility [19].

At birth, the original squamocolumnar junction (SCJ) is typically located on the ectocervix, reflecting the outward extension of Müllerian columnar epithelium during fetal development [21]. Postnatally, this position is not fixed but undergoes dynamic remodeling driven by hormonal and metaplastic processes. During childhood, in the low-estrogen environment, the SCJ tends to recede toward the endocervical canal. With the onset of puberty and the associated rise in estrogen, cervical eversion (ectropion) occurs, exposing columnar epithelium on the ectocervix and shifting the SCJ outward once again; this newly exposed columnar epithelium then undergoes squamous metaplasia, forming the transformation zone [22]. This metaplastic process is driven largely by subcolumnar reserve cells, which proliferate and differentiate into immature and then mature squamous epithelium, progressively displacing the SCJ toward the external os [23]. Pregnancy and use of hormonal contraception further promote eversion and enlargement of the transformation zone, while the menopausal decline in estrogen causes the SCJ to retract into the endocervical canal, often rendering it non-visible on colposcopic examination [24]. This centripetal migration of the SCJ with age has direct clinical relevance, as a non-visible or receding SCJ is associated with a higher likelihood of missing high-grade squamous lesions on cytology and colposcopy [24]. Because the transformation zone and the immature metaplastic epithelium at the SCJ are particularly vulnerable to HPV infection and viral persistence [25], the lifelong repositioning of this junction—from fetal exposure, through pubertal eversion, to postmenopausal retraction—defines a shifting anatomical substrate for hormone-modulated carcinogenesis that is central to the arguments developed in this review.

## 3. ER/PR Changes in Epithelium and Stroma During the Normal Menstrual Cycle

The ectocervix is a dynamic hormone-responsive tissue, and its cellular and molecular properties fluctuate throughout the menstrual cycle in coordination with systemic estradiol (E2) and progesterone (P4) levels [26]. Both ERα and progesterone receptor (PR) are expressed in the epithelial and stromal compartments of the cervix. Still, their levels, distribution, and transcriptional activity exhibit significant compartment-specific variations that are closely linked to hormonal changes [10].

During the follicular (proliferative) phase, rising E2 levels bind to and activate ERα in ectocervical stromal cells. This activation triggers the paracrine release of growth factors, including insulin-like growth factor 1 (IGF1), fibroblast growth factors (FGFs), and Wnt ligands, which stimulate the proliferation of the overlying epithelium [27]. Concurrently, E2-liganded ERα upregulates PR expression in both stromal and epithelial cells, ensuring effective transduction of the subsequent P4 surge [13]. Immunohistochemical analyses show that ERα staining is detectable in the basal and parabasal layers of the ectocervical epithelium during the proliferative phase, while stromal cells exhibit more diffuse ERα positivity [13].

Following ovulation, the luteal (secretory) phase is characterized by elevated P4 levels that activate PR, which in turn downregulates ERα expression and reverses E2-induced proliferative changes [28]. PR activation in the cervical epithelium promotes differentiation and mucinification, with increased mucus secretion from endocervical glands serving as a hallmark P4 response [10]. Both PR isoforms—PRA and PRB, transcribed from a single *PGR* gene via alternative promoters—are upregulated in the cervical epithelium during the secretory phase, although PRB expression is particularly prominent in stromal cells [29]. Notably, ERβ expression appears limited compared with ERα in cervical tissues, although its contribution to cervical physiology remains less clearly defined [29].

Disruption of this balance—due to sustained high E2 without adequate P4 opposition, as seen with chronic oral contraceptive use, anovulatory cycles, or obesity-related hyperestrogenism—may create conditions conducive to HPV-driven neoplastic transformation [6]. Moreover, endocrine-disrupting chemicals (EDCs) that mimic or antagonize estrogen signaling can disrupt this cycle, potentially influencing cervical cancer susceptibility [30].

Earlier studies employing steroid binding-site assays in human cervical tissue across the full ovarian cycle similarly documented cyclic fluctuation in estrogen- and progestogen-binding capacity, with a nadir coinciding with the menstrual phase, consistent with the proliferative-to-secretory pattern described above [31,32].

Compartment-specific ERα and PR expression across the menstrual cycle in the normal ectocervix is listed in Table 1.

## 4. Relation of Sex Hormones and Ectocervical SCC

The relationship between sex hormones and cervical cancer has been explored through various epidemiological approaches. A landmark collaborative reanalysis of 24 studies involving over 16,000 cases confirmed that current use of combined oral contraceptives containing E2 and synthetic progestins increases cervical cancer risk in a duration-dependent manner, with risk declining after cessation [33]. Women with high parity—a condition of prolonged elevated E2 and P4 during pregnancy—also exhibit a significantly increased risk, even after controlling for other cofactors [34].

Studies measuring serum hormone levels further support the influence of hormones. A prospective study within the European Prospective Investigation into Cancer and Nutrition cohort demonstrated that elevated circulating E2 levels are positively associated with cervical cancer risk [35]. Similarly, elevated 16α-hydroxyestrone, a genotoxic E2 metabolite, has been found at higher concentrations in women with HPV-associated cervical neoplasia [36]. These genotoxic estrogen metabolites can cause DNA adducts and oxidative damage in cervical epithelial cells, potentially accelerating the accumulation of somatic mutations necessary for invasive cancer [37].

The mechanistic interplay between estrogen and HPV oncogenes has been particularly illuminated by studies using the K14-HPV16/18 transgenic mouse model. In these animals, exogenous E2 is required for the development of cervical intraepithelial neoplasia (CIN) and invasive squamous cell carcinoma; without estrogen, even persistent HPV oncogene expression is insufficient to drive carcinogenesis [30]. Cessation of estrogen treatment results in the regression of established neoplastic lesions, while estrogen antagonists—including selective estrogen receptor modulators (SERMs) such as tamoxifen and raloxifene—prevent lesion formation and promote regression of existing disease [38]. These findings establish a causal rather than merely associative role for estrogen in HPV-mediated cervical carcinogenesis.

In contrast, the relationship between progesterone and ectocervical SCC risk is more complex and context-dependent. Several case–control studies found no significant association between depot medroxyprogesterone acetate use and cervical cancer risk, while others reported a modest elevation [39]. These discrepancies likely reflect failure to stratify by HPV status, confounding by sexual behavior, and the dual roles of PR isoforms in different tissue compartments [40]. Experimental evidence from mouse models demonstrates that P4-PR signaling in the cervical epithelium has a tumor-suppressive function, whereas the stromal PR axis appears to limit invasive spread [41]. Collectively, these data support a model in which the E2/ERα axis is predominantly pro-tumorigenic, while the P4/PR axis is predominantly protective, with the ultimate phenotypic outcome depending on the cellular compartment, receptor isoform, and hormonal context [42].

## 5. The Carcinogenic Role of Ectocervical Stromal ERα

The discovery that ERα expression is progressively lost from cervical epithelial cells during carcinogenesis, while being retained in stromal cells, has fundamentally reframed our understanding of estrogen’s role in cervical cancer biology [43]. Early immunohistochemical studies found that ERα was detectable in normal ectocervical epithelium but largely absent from SCC, leading to the initially puzzling conclusion that estrogen-responsive cells were not the direct targets of carcinogenic estrogen action [43].

The resolution of this paradox came from landmark mouse model studies demonstrating that stromal ERα is both necessary and sufficient for estrogen-mediated cervical carcinogenesis. Chung and colleagues showed that germline ERα knockout rendered K14E7 transgenic mice resistant to estrogen-induced cervical neoplasia [44]. Crucially, temporal deletion of ERα specifically in cervical stromal cells—using a tamoxifen-inducible Cre recombinase system—resulted in the complete regression of established CIN3 lesions and dramatic reductions in epithelial cell proliferation, despite intact epithelial ERα expression [27]. Conversely, epithelial-specific ERα deletion had no significant effect on neoplastic disease, confirming that stromal ERα is the primary driver of estrogen-dependent cervical carcinogenesis [45].

The molecular mechanism underlying this stromal-to-epithelial paracrine oncogenic signaling has been partially elucidated. Activated ERα then upregulates the expression of paracrine-acting growth factors and cytokines—including IGF1 [46], FGFs [47], Wnt ligands [48], CXCL12 [49,50], and IL-8 [51]—that are secreted into the tumor microenvironment, exerting mitogenic, pro-angiogenic, and pro-inflammatory effects on the adjacent HPV-positive epithelium. Genome-wide transcriptomic profiling of laser-capture microdissected cervical stroma from estrogen-treated HPV transgenic mice identified a unique estrogen-responsive gene expression signature enriched for chemokine/cytokine activity and inflammatory immune functions associated with carcinogenesis [52].

Human cervical cancer data align with these murine findings. An analysis of gene expression across normal cervix, CIN1, CIN2/3, and invasive SCC revealed a progressive, stepwise depletion of ERα expression in the cervical epithelium that correlated remarkably with histopathological grade [12]. The inverse correlation between epithelial ERα loss and p16INK4a expression—a well-established surrogate for high-risk HPV activity—suggests that HPV oncoproteins E6 and E7 actively drive the epigenetic silencing of ERα in the transformation zone epithelium, possibly through polycomb repressor complex activation [12]. Meanwhile, stromal fibroblasts and CAFs retain ERα expression, and ex vivo cultures of cervical CAFs demonstrate E2-responsive ERα signaling [53]. Our group’s IHC analysis of 95 cervical carcinoma specimens confirmed that ERα was predominantly expressed in stromal rather than carcinoma cells, with higher stromal ERα correlating with earlier International Federation of Gynecology and Obstetrics (FIGO) stages and superior survival outcomes [13]. A subsequent larger cohort study of 169 specimens further validated that stromal ERα expression correlated inversely with hematogenous distant metastasis rates, providing clinical evidence that the stromal ERα axis constrains tumor aggressiveness at later stages of disease [54].

In summary, stromal ERα functions as a nexus that integrates systemic estrogenic signals with HPV-driven epithelial oncogenesis, orchestrating a permissive tumor microenvironment through paracrine growth factor secretion.

Stromal ERα-driven paracrine factors implicated in cervical carcinogenesis are listed in Table 2.

## 6. The Anti-Transformation Role of Ectocervical Epithelial PR

While epithelial ERα is progressively silenced during cervical carcinogenesis, the PR in cervical epithelial cells serves as a critical tumor suppressor. The anti-transformative function of epithelial PR is supported by convergent evidence from mouse models, human tissue analyses, and genomic databases [55].

The HPV transgenic mouse model has been particularly informative. In K14E6/K14E7 mice, short-term treatment with medroxyprogesterone acetate (MPA), a synthetic progestin with high affinity for PR, prevents established CIN lesions from progressing to invasive cancer [28]. When transgenic mice bearing CIN lesions were treated with MPA, cervical cancer was absent in the treated group but developed in all vehicle-treated controls. This preventive effect required PR expression in cervical epithelial cells: MPA was completely ineffective in K14E7/Pgr−/− mice lacking PR, while partial protection was maintained in Pgr+/− haploinsufficient mice, demonstrating that PR is a haploinsufficient tumor suppressor in the cervix [56].

The tumor-suppressive mechanism of epithelial PR involves inhibiting proliferation and inducing differentiation. MPA treatment promotes mucinification—a form of terminal differentiation—in cervical cancer cells in an alcian blue-positive, PR-dependent manner [55]. Bromodeoxyuridine incorporation assays demonstrate that MPA significantly reduces epithelial cell proliferation rates, while flow cytometry analyses confirm the induction of apoptosis in PR-positive but not PR-negative cervical cancer cells [28]. Transcriptomic analysis of MPA-treated cervices identified upregulation of Myc target genes involved in terminal keratinocyte differentiation and downregulation of cell cycle progression genes, suggesting that epithelial PR promotes exit from the proliferative state [10].

Human tissue and genomic data further support the tumor-suppressive role of epithelial PR. IHC analyses consistently demonstrate that PR expression is significantly lower in squamous cell carcinoma and adenocarcinoma of the cervix compared with normal cervical epithelium [11,57]. Analysis of The Cancer Genome Atlas cervical cancer dataset reveals that *PGR* gene expression is reduced in tumor epithelium relative to adjacent normal tissue, and that reduced PR expression correlates with worse clinical outcomes [56]. Most importantly, PR is undetectable in the majority of spontaneously arising cancers in HPV transgenic mouse models—even in mice with only one deleted *Pgr* allele—indicating that selective pressure during carcinogenesis drives PR loss as a mechanism of immune and hormonal escape [56].

Critically, estrogen attenuates the tumor-suppressive efficacy of MPA. In mice treated with combined E2 and MPA, cancer regression was significantly blunted compared with MPA-only treatment, and this antagonism operated specifically through the epithelial compartment [58]. This finding has profound clinical implications: progestin-based chemoprevention or therapy of CIN lesions is likely to be most effective in the context of low ambient estrogen levels, suggesting that co-administration of estrogen antagonists or SERMs may potentiate PR-mediated tumor suppression [42].

## 7. The Antimetastatic Role of Ectocervical Stromal PRB

Beyond its well-characterized role in epithelial tumor suppression, PR—particularly the PRB isoform—in the cervical stroma plays a significant antimetastatic role. PRB and PRA are encoded by a single *PGR* gene but differ structurally due to the presence of an additional N-terminal activation function domain (AF-3) in PRB. This difference renders the two isoforms functionally distinct transcription factors that regulate overlapping, yet non-identical, target gene sets [59].

Our group’s initial cohort study of 95 cervical SCC specimens [13] reported that PRB, like ERα, was predominantly expressed in stromal rather than carcinomatous epithelial cells. Kaplan–Meier analyses demonstrated that higher stromal PRB expression correlated with improved 5-year overall survival. Multivariable Cox regression confirmed that stromal PRB was an independent prognostic indicator after adjusting for FIGO stage, histologic grade, and lymphovascular space invasion [13]. Notably, stromal ERα and PRB expression were highly correlated, suggesting that a shared regulatory mechanism maintains both receptors in the cervical stroma.

An extended cohort study of 169 cervical SCC specimens provided further mechanistic insight by specifically linking stromal PRB to the prevention of hematogenous metastasis [54]. Patients with higher stromal PRB expression had significantly lower rates of hematogenous distant metastasis (*p* = 0.011), and stromal ERα also correlated with reduced hematogenous spread (*p* = 0.013). Multivariable logistic regression confirmed that stromal PRB independently reduced the 5-year mortality risk (*p* = 0.022), regardless of standard clinicopathological parameters [54]. Importantly, incorporating stromal PR (A + B) and PRB into FIGO staging significantly improved the accuracy of survival predictions, suggesting that stromal PR status provides clinically meaningful prognostic information beyond current staging systems.

The mechanism by which stromal PRB suppresses hematogenous metastasis likely involves paracrine modulation of tumor cell invasiveness and vascular remodeling [54]. In the broader context of gynecologic cancer, stromal-epithelial PRB signaling has been linked to the downregulation of matrix metalloproteinases (MMPs), upregulation of tissue inhibitors of metalloproteinases, and suppression of epithelial-to-mesenchymal transition (EMT) mediators [60]. Progesterone receptor activation in stromal fibroblasts may also decrease the secretion of pro-invasive paracrine factors such as TGF-β, VEGF, and CXCL12, limiting the formation of a pre-metastatic niche in distant organs [61]. In the specific context of hematogenous spread—which is relatively uncommon in early-stage SCC but portends a dismal prognosis when it occurs—stromal PRB may restrict the intravasation of tumor cells by preserving basement membrane integrity and maintaining the differentiated phenotype of peritumoral stromal cells [61].

**Figure 1:**
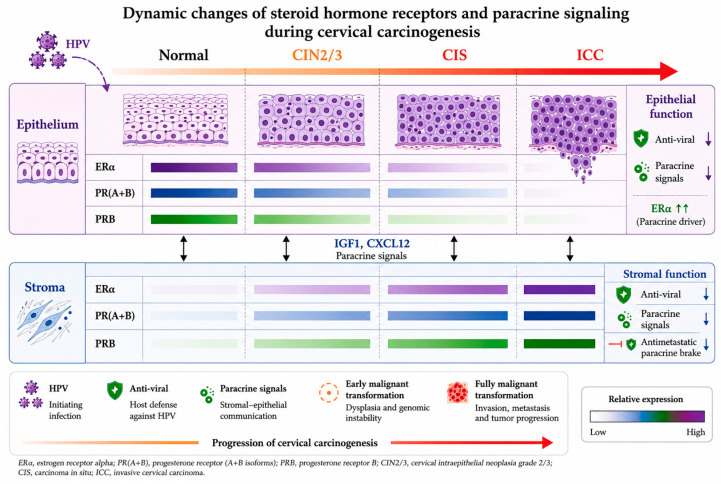
Proposed model of dynamic changes in steroid hormone receptor expression and paracrine signaling during cervical carcinogenesis. Schematic illustration of the dynamic alterations in epithelial and stromal steroid hormone receptor expression during the progression of cervical carcinogenesis from normal cervical epithelium to cervical intraepithelial neoplasia grade 2/3 (CIN2/3), carcinoma in situ (CIS), and invasive cervical carcinoma (ICC). Persistent human papillomavirus (HPV) infection initiates cervical carcinogenesis and is accompanied by progressive disruption of epithelial–stromal communication. In the epithelial compartment, the expression of estrogen receptor alpha (ERα), progesterone receptor (PR; PR-A + B), and progesterone receptor B (PRB) gradually decreases with disease progression, concomitant with reduced antiviral activity and impaired paracrine signaling. In contrast, stromal ERα, PR (A + B), and PRB expression progressively increases, suggesting activation of a tumor-supportive stromal microenvironment and enhanced stromal responsiveness to hormonal signaling. Elevated stromal ERα is associated with increased production of paracrine mediators, including IGF1 and CXCL12, which promote epithelial proliferation and malignant progression. Meanwhile, stromal PRB functions as an antimetastatic paracrine brake that counteracts tumor dissemination, although this protective effect becomes insufficient during advanced disease. Together, these reciprocal alterations in epithelial and stromal hormone receptor expression highlight the critical role of epithelial–stromal crosstalk in cervical cancer development and progression. Abbreviations: ERα, estrogen receptor alpha; PR (A + B), progesterone receptor A + B isoforms; PRB, progesterone receptor B; HPV, human papillomavirus; CIN2/3, cervical intraepithelial neoplasia grade 2/3; CIS, carcinoma in situ; ICC, invasive cervical carcinoma; IGF1, insulin-like growth factor 1; CXCL12, C-X-C motif chemokine ligand 12. ↓: decrease, ↑: increase, ↕: interaction.

The topological analysis of sex hormone receptor expression further contextualized these findings within the broader landscape of cervical SCC management [7], which increasingly incorporates IHC-based receptor testing alongside PD-L1 assessment for treatment stratification [62]. This convergence underscores the clinical readiness of stromal PR assessment as a companion diagnostic, potentially enabling the identification of patients who may benefit from progestin-based adjuvant therapies or who require more aggressive surveillance for hematogenous relapse (Figure 1).

**Figure 2:**
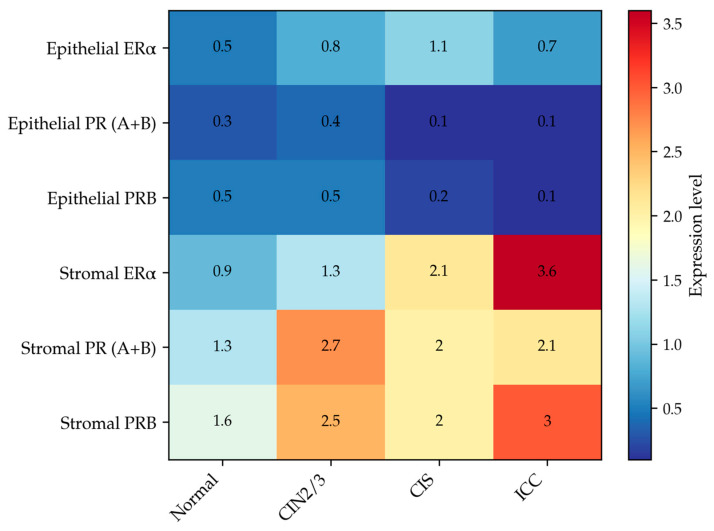
Heatmap of compartment-specific estrogen receptor alpha (ERα) and progesterone receptor (PR) expression across cervical carcinogenesis. Mean immunoreactive scores (IRS), calculated as staining intensity (0–3) multiplied by percentage of positive cells (0–4), for ERα, PR (A + B), and PRB in the epithelial and stromal compartments are shown across normal cervical tissue, cervical intraepithelial neoplasia grades 2/3 (CIN2/3), carcinoma in situ (CIS), and invasive cervical carcinoma (ICC). Color intensity scales with IRS magnitude (pale yellow = low, deep red = high). All IRS values are derived directly from the immunohistochemical cohort data reported in Hong et al. [7]; no new quantification, restaining, or reanalysis was performed for this figure.

Figure 2 summarizes these compartment- and stage-specific expression trends as a heatmap, integrating the quantitative immunoreactive score (IRS) data described above across the normal-to-ICC continuum [7,54].

## 8. Future Directions

The mechanistic framework reviewed herein—where stromal ERα drives carcinogenic paracrine signaling, epithelial PR suppresses neoplastic transformation, and stromal PRB limits hematogenous metastasis—opens multiple clinically actionable avenues for research and therapeutic development [7].

First, the development and validation of standardized IHC assays for stromal ERα and PRB as companion diagnostics is a near-term priority [63]. Current cervical cancer staging relies exclusively on clinicopathological parameters, and the independent prognostic value of stromal receptor expression demonstrated in our cohorts warrants prospective validation in multicenter studies across diverse populations [7]. Integrating stromal PR status into FIGO staging or risk stratification algorithms could help identify women at the highest risk of hematogenous relapse who would benefit from intensified surveillance or adjuvant systemic therapy [7]. Prospective multicentre validation is required before stromal hormone receptor assessment can be incorporated into clinical risk stratification.

Second, clinical trials evaluating SERMs—such as tamoxifen and raloxifene, which are already in widespread clinical use for breast cancer—in women with high-grade CIN (CIN2/3) and HPV persistence represent a logical translational step [64]. Mouse model data showing that SERM therapy induces regression of established CIN lesions and prevents recurrence provide a compelling preclinical rationale [41]. Crucially, trial designs should stratify participants by HPV status and PR expression to identify the subpopulation most likely to benefit—namely, HPV-positive women with retained epithelial PR expression and evidence of stromal ERα activity [41].

Third, the concept of PR status-guided progestin chemoprevention deserves prospective investigation. Given that MPA regresses CIN in PR-positive but not PR-negative mouse tumors, and that estrogen antagonizes this protective effect, a rational strategy for HPV-positive women with CIN2/3 and low ambient estrogen would combine depot medroxyprogesterone acetate or oral progestins with an anti-estrogen agent [65]. Biomarkers for PR activity—rather than simple protein expression—need to be developed, as receptor activity is determined by both expression levels and ligand availability in the context of cyclical hormonal fluctuations [66].

Fourth, the molecular identification of ERα-regulated paracrine factors secreted by cervical stromal CAFs represents a rich source of therapeutic targets. Candidates, including CXCL12, IL-8, and IGF1, could be targeted by small molecules or biologic agents to disrupt the pro-carcinogenic stromal-epithelial signaling axis [67]. Such targeted interventions might complement HPV vaccination and screening programs, particularly for women who are already HPV-positive and harbor high-grade CIN [68].

Fifth, the role of EDCs in modulating cervical ERα and PR activity warrants further epidemiological and experimental investigation [69]. Given the global burden of EDC exposure through agricultural, industrial, and consumer sources, characterizing their effect on cervical cancer risk—particularly in combination with HPV infection—is a critical public health research priority [70].

Sixth, future studies should examine whether menopause, hormone therapy, and polycystic ovary syndrome—all conditions that alter the E2/P4 ratio—differentially affect cervical cancer risk through modulation of stromal ERα and epithelial PR activity.

Finally, integrating single-cell RNA sequencing, spatial transcriptomics, and organoid technology offers unprecedented opportunities to dissect the cellular heterogeneity of cervical ERα and PR signaling at single-cell resolution [71]. Cervical organoid models that recapitulate the transformation zone architecture could serve as platforms for testing SERM and selective progesterone receptor modulator efficacy ex vivo, accelerating the pipeline from mechanistic discovery to clinical application [72]. Collectively, these research directions position sex hormone receptor biology as a central pillar of next-generation cervical cancer diagnostics and therapeutics.

## 9. Conclusions

The malignant transformation of the uterine ectocervical SCC is not solely the product of viral oncogenesis but emerges from a complex interplay between persistent high-risk HPV infection and a dysregulated sex hormone receptor microenvironment. The evidence reviewed herein converges on a topologically defined model in which stromal ERα functions as a pro-carcinogenic paracrine amplifier, epithelial PR serves as a ligand-dependent tumor suppressor constraining neoplastic progression, and stromal PRB confers antimetastatic protection by limiting hematogenous dissemination. This compartment-specific receptor biology reflects the embryological blueprint of the Müllerian duct, where stromal signals have always governed overlying epithelial behavior, and underscores the necessity of interpreting receptor expression in its spatial context rather than as a global tissue property. Clinically, these insights reframe sex hormone receptor assessment from a rudimentary adjunct to a mechanistically grounded biomarker strategy: stromal PRB expression has shown prognostic associations in retrospective studies and requires prospective validation, while epithelial PR status may ultimately guide the selection of patients most likely to benefit from progestin-based chemoprevention of high-grade CIN. As cervical cancer management enters an era of precision diagnostics incorporating PD-L1 scoring, molecular subtyping, and targeted therapeutics, the systematic integration of stromal ERα and PRB immunohistochemistry into clinical decision-making represents a near-term, cost-effective, and biologically rational advance. Future prospective studies, organoid-based drug screening platforms, and clinical trials of SERMs and selective progesterone receptor modulators will be essential to translate these mechanistic discoveries into tangible reductions in cervical cancer morbidity and mortality worldwide.

## Figures and Tables

**Table 1:** Compartment-specific ERα and PR expression across the menstrual cycle in the normal ectocervix.

Cycle Phase	Compartment	ERα	PR (A + B)	Key Functional Notes	Refs
Proliferative (follicular)	Epithelium	Detectable in basal/parabasal layers	Upregulated (E2-ERα–driven)	Prepares epithelium for subsequent P4 response	[13,27]
Proliferative (follicular)	Stroma	Diffusely positive, activated by rising E2	Upregulated	Drives paracrine release of IGF1, FGFs, Wnt ligands, stimulating epithelial proliferation	[13,27]
Secretory (luteal)	Epithelium	Downregulated by PR activation	Both isoforms upregulated	PR activation promotes differentiation and mucinification	[10,28,29]
Secretory (luteal)	Stroma	Downregulated	PRB isoform particularly prominent	P4-PR counteracts E2-driven proliferation	[28,29]
Menstrual	Epithleium/Stroma	Nadir, paralleling falling serum E2	Nadir, paralleling falling serum P4	Classical binding-site studies in cervical tissue across the full ovarian cycle (including menses) documented cyclic steroid receptor/binding-site fluctuation broadly consistent with the proliferative-to-secretory pattern above, though compartment-resolved (epithelium vs. stroma) IHC data specific to the menstrual phase remain sparse relative to the follicular and luteal phases	[31,32]

Abbreviation: ERα, estrogen receptor alpha; PR, progesterone receptor; PR (A + B), progesterone receptor isoforms A and B combined; PRB, progesterone receptor isoform B; E2, estradiol; P4, progesterone; IGF1, insulin-like growth factor 1; FGFs, fibroblast growth factors; IHC, immunohistochemistry.

**Table 2:** Stromal ERα-driven paracrine factors implicated in cervical carcinogenesis.

Factor	Putative Receptor	Downstream Pathway	Reported Oncogenic Function	Refs
IGF1	IGF1R	PI3K/AKT, MAPK	IGF-1/IGF-1R signaling stimulates cervical cancer cell invasiveness and proliferation, modulated by αvβ3 integrin	[46]
FGFs (incl. KGF/FGF7)	FGFR2IIIb	RAS/ERK	KGFR (FGFR2 IIIb) is expressed in human uterine cervical cancer	[47]
Wnt ligands	Frizzled/LRP	Canonical Wnt/β-catenin	Wnt–β-catenin pathway activation in basal-parabasal layers of normal cervical epithelium, sustained through cervical carcinoma development	[48]
CXCL12 (SDF-1)	CXCR4	Chemokine/GPCR signaling	Pro-inflammatory and pro-angiogenic; chemokine-driven microenvironment remodeling	[49,50]
IL-8	CXCR1/CXCR2	NF-κB, MAPK	IL-8 expression correlates with angiogenesis (microvessel/macrophage counts) and poor prognosis in uterine cervical cancer	[51]

Abbreviation: IGF1, insulin-like growth factor 1; IGF1R, insulin-like growth factor 1 receptor; PI3K/AKT, phosphoinositide 3-kinase/protein kinase B; MAPK, mitogen-activated protein kinase; FGFs, fibroblast growth factors; KGF/FGF7, keratinocyte growth factor/fibroblast growth factor 7; FGFR2IIIb, fibroblast growth factor receptor 2 IIIb; RAS/ERK, rat sarcoma/extracellular signal-regulated kinase; LRP, low-density lipoprotein receptor-related protein; CXCL12, C-X-C motif chemokine ligand 12; SDF-1, stromal cell-derived factor 1; CXCR4, C-X-C motif chemokine receptor 4; GPCR, G-protein-coupled receptor; IL-8, interleukin-8; CXCR1/CXCR2, C-X-C motif chemokine receptor 1/2; NF-κB, nuclear factor kappa-light-chain-enhancer of activated B cells.

## Data Availability

No new data were created or analyzed in this study. Data sharing is not applicable to this article.

## Abbreviations

The following abbreviations are used in this manuscript:

BMP	Bone morphogenetic protein
CAFs	Cancer-associated fibroblasts
CIN	Cervical intraepithelial neoplasia
CxCa	Cervical cancer
E2	Estradiol
EDCs	Endocrine-disrupting chemicals
EMT	Epithelial-to-mesenchymal transition
ERα	Estrogen receptor alpha
ERβ	Estrogen receptor beta
FGFs	Fibroblast growth factors
FIGO	International Federation of Gynecology and Obstetrics
HPV	Human papillomavirus
ICC	Invasive cervical carcinoma
IGF1	Insulin-like growth factor 1
IHC	Immunohistochemistry
IL-8	Interleukin-8
MPA	Medroxyprogesterone acetate
P4	Progesterone
PD-L1	Programmed death-ligand 1
PR	Progesterone receptor
PRA	Progesterone receptor isoform A
PRB	Progesterone receptor isoform B
SERMs	Selective estrogen receptor modulators
SCC	Squamous cell carcinoma
SPRMs	Selective progesterone receptor modulators
TGF-β	Transforming growth factor beta
VEGF	Vascular endothelial growth factor
